# Craniopharyngioma, Chronotypes and Metabolic Risk Profile

**DOI:** 10.3390/nu13103444

**Published:** 2021-09-28

**Authors:** Carolina Di Somma, Elisabetta Scarano, Luigi Barrea, Domenico Solari, Enrico Riccio, Rossana Arianna, Luigi Maria Cavallo, Fiammetta Romano, Elea Di Benedetto, Alice Rodriguez, Giulia de Alteriis, Annamaria Colao

**Affiliations:** 1 Endocrinology Unit, Department of Clinical Medicine and Surgery, Federico II University, 80131 Naples, Italy; el.scarano@gmail.com (E.S.); luigi.barrea@unipegaso.it (L.B.); enr.riccio@gmail.com (E.R.); rossanarianna@gmail.com (R.A.); fromano.med@gmail.com (F.R.); dibenedettoelea@gmail.com (E.D.B.); ali.9@live.it (A.R.); dealteriisgiulia@gmail.com (G.d.A.); colao@unina.it (A.C.); 2 Cattedra Unesco “Educazione Alla Salute e allo Sviluppo Sostenibile”, Federico II University, 80131 Naples, Italy; 3 Department of Neurosciences, Reproductive and Odontostomatological Sciences, Federico II University, 80125 Naples, Italy; domenico.solari@unina.it (D.S.); luigimaria.cavallo@unina.it (L.M.C.)

**Keywords:** pituitary neoplasm, hypothalamus, craniopharyngioma, obesity, chronotype

## Abstract

Aim: To investigate the potential association among Craniopharyngioma (CP), chronotypes and metabolic risk profile. Subjects and Methods: The study population included 28 patients (46.4% males; 42.6 ± 15.8 years) and 28 controls, age, gender and BMI matched (46.4% males; 46.5 ± 12.9 years). In this study sample, we evaluated: anthropometric measurements (waist circumference, WC; BMI), plasma glucose, lipid profile, and systolic (SBP) and diastolic (DBP) blood pressure. Morningness-Eveningness was measured with the Horne-Ostberg Morningness-Eveningness Questionnaire (MEQ), which included 19 questions about preferred sleep time and daily performance. Results: in both patients and controls grade I obesity was detected in 15 subjects (53.6%), grade II obesity in 13 subjects (46.4%). In the patient group, the mean score of chronotype was 47.8 ± 12.6. In particular, 9 patients (32.1%) exhibited the morning chronotype, 6 (21.4%) the intermediate chronotype and 13 (46.4.%) the evening chronotype. No significant difference was found in gender and age among the chronotype categories. Patients with the evening chronotype had higher blood pressure values and worse metabolic parameters than those with the morning chronotype. In the control group, the mean score of the chronotype was 57.6 ± 9.5. In particular, 16 (57.1%) subjects exhibited the morning chronotype, 10 (35.7%) the intermediate chronotype and only 2 (7.1.%) the evening chronotype. The prevalence of intermediate and evening chronotypes was higher in females than males (*p* = 0.021), while males have a higher prevalence of the morning chronotype. Subjects with intermediate and evening chronotypes had worse metabolic parameters than those with the morning chronotype. In patients, the chronotype score was inversely correlated to WC, BMI, SBP, DBP, plasma glucose, total cholesterol, triglycerides, LDL cholesterol and positively correlated with HDL cholesterol. No correlation was found between age and chronotype. In controls, the chronotype score was inversely correlated to WC, BMI, plasma glucose, total cholesterol, LDL cholesterol. No correlation was found among chronotype and age, blood pressure, triglycerides, HDL cholesterol. Considering the whole population of the study (patients and controls), at logistic regression the chronotype score was significantly associated with the presence of CP. Conclusions: for the first time thus far, our study puts the light on the association of the CP with chronotypes and metabolic alterations in this disease, which are the main determinants of the reduced quality of life, higher morbidity and mortality in this setting of patients. This finding suggests that alterations of chronotype might represent an adjunctive risk for CP patients and a possible target for their integrate management.

## 1. Introduction

Craniopharyngioma (CP) is an uncommon epithelial intracranial tumour that can arise anywhere along the path of the craniopharyngeal duct, although the majority arises in the sellar/parasellar region, predominantly in the suprasellar space [1,2]. Despite its benign histological morphology, the critical relationship with surrounding vascular and nervous structures may hamper the definitive treatment of CP and often causes unfavourable long-term sequelae after surgical removal [3]. In epidemiological studies, CPs account for approximately 1% of all primary intracranial tumours [3,4]. Surgery is presently the recommended first therapeutic approach, combined or not with adjuvant radiotherapy [5,6].

Among the particular challenges that CP presents, the risk of obesity due to hypothalamic derangement is one of the most demanding and implies devastating consequences for patients. Indeed, the increased risk of long-term mortality appears to be associated with hypothalamic dysfunction and related manifestations [7,8]. There is a lot of evidence that tumours with hypothalamic involvement confer a high risk of obesity [9,10] and that the degree of this involvement is correlated with the severity of the postoperative obesity [11,12]. Obesity from hypothalamic injury is thought to result from damage of the regions involved in the central control of feeding and circadian rhythm synchronisation [13]. Weight gain occurs despite the adequate endocrine replacement of pituitary hormone deficiencies [14,15]. As a consequence, the most compelling challenge is that obesity in patients with CP is mainly unresponsive to lifestyle intervention that has proved to be effective in simple obesity, with increased metabolic morbidities and cardiovascular mortalities [16,17]. Actually, patients often show marked weight gain even after forced caloric restriction, suggesting an overall decrease in energy expenditure. Several drugs have been attempted to treat obesity in patients with CP, but an effective pharmacotherapy is still lacking. Bariatric surgical procedures may play an important role for weight loss in patients with CP, but evidence of its effectiveness and safety is still scanty.

Treatment of obesity in patients with CP still remains an unmet need. Nevertheless, recent research in the pathophysiological mechanisms may open the way for a more effective treatment of obesity in these patients to improve their long-term prognosis and quality of life. 

A growing body of evidence suggests the roles of chronotype in the development of obesity [18]. There are three types of chronotype, based on the circadian behavioural phenotypes: morning, evening and intermediate chronotypes [19]. Subjects characterised by the morning chronotype rise early and prefer activities earlier in the day, while those with the evening chronotype prefer later bed and wake times and are more inclined to have their time peak activity later in the day. Intermediate chronotype falls between the two. Of interest, the evening chronotype has a propensity to higher morbidity and mortality compared to the morning chronotype [20]. In particular, individuals with the evening chronotype have a lower adherence towards a healthy diet [21,22] and are more prone to develop obesity and metabolic diseases, such as type 2 diabetes and metabolic syndrome, compared to those with a morning or intermediate chronotype [20,23,24,25]. Thus, chronotype and circadian rhythms are emerging risk factors of obesity and cardiometabolic diseases [21]. Despite the large body of evidence linking the evening type with higher obesity risk, differences in chronotypes in obese CP patients have not reported previously, and consequently, there is no knowledge on whether the peculiar injury of the hypothalamic regions in CP could be involved in different chronotypes between obese patients with and without CP. 

To the best of our knowledge, no data were reported on adult patients with CP to examine the contribution of chronotype to obesity and metabolic alterations in this setting. Considering the possible link between CP and the synchronisation of circadian rhythm and the scant results of the available anti-obesity therapeutics in patients with CP, the aim of the study was the potential association among CP, chronotypes and metabolic risk profile compared with a population of BMI-matched controls.

## 2. Subjects and Methods

This is an observational case-control study. The study was approved by the Federico II Ethical Committee with protocol number *n* = 223/19 and was carried out in accordance with the Declaration of Helsinki. 

### 2.1. Subjects

#### 2.1.1. Patients

Patients treated for CP were consecutively recruited from the Department of Endocrinology, AOU Federico II Napoli Italy from January 2019 to December 2020. CP was diagnosed from 2001 to 2020. The inclusion criterion was being a patient aged 18–70 who had previously been treated for CP. Exclusion criteria were: insufficient substitution of pituitary hormone deficiencies within 6 months prior to inclusion, epileptic seizures and alcohol or drug abuse. The final study population consisted of 28 patients (46.4% males; 42.6 ± 15.8 years). The characteristics of patients with CP at study entry are shown in Table 1.

#### 2.1.2. Controls

To avoid the confounding effects of age, sex and BMI, we included 28 controls matched for age, sex and BMI among 172 subjects that were consecutively enrolled in a campaign to prevent obesity called the OPERA (obesity, programs of nutrition, education, research and assessment of the best treatment) Prevention Project [26,27]. Subjects gave their written informed consent to the study that was carried out in agreement with the Helsinki declaration for human studies. Eligible participants for the study were subjects having normal liver, cardiopulmonary and kidney functions as determined by self-reported medical history. In addition, we excluded subjects taking medications for hepatic, renal and cardiopulmonary diseases. Other exclusion criteria were alcohol abuse assessed as previously reported [23] and/or drug abuse assessed performing a thorough evaluation. Subjects on a specific dietary regimen for any reason were also excluded from the study. No subject was taking sleeping pills or central nervous system drugs. 

### 2.2. Anthropometric Parameters

Subjects dressed in light clothes only and took their shoes off when anthropometric parameters were assessed, as already reported [27]. Body mass Index (BMI) was calculated as weight (kg)/height (m^2^). Body weight was assessed with a calibrated scale. A wall-mounted stadiometer was used to assess height. Waist circumference (WC) was evaluated to the closest 0.1 cm with a non-extensible tape [28]. Grade I Obesity was defined if BMI ranged from 30 to 34.9 kg/m^2^, grade II obesity if BMI ranged from 35 to 39.9 kg/m^2^ WHO|World Health Organisation. Body Mass Index—BMI. Available online: https://www.euro.who.int/en/health-topics/disease-prevention/nutrition/a-healthy-lifestyle/body-mass-index-bmi (accessed on 24 September 2021).

Hypopituitarism was diagnosed clinically based on the clinical manifestations, the baseline assessment of pituitary function and stimulus test, according to the Clinical Practice Guidelines of the Endocrine Society [29]. 

Peripheral venous blood samples were taken in the morning between 8 and 10, after fasting for at least 8 h and conserving at −80 °C until processing. All biochemical analyses, including fasting plasma glucose, total cholesterol, fasting plasma triglycerides, were performed with the standard methods of the central biochemistry laboratory of our Institute. The low-density lipoprotein (LDL) and high-density lipoprotein (HDL) cholesterol were determined by a direct method (homogeneous enzyme test for direct quantitative determination of LDL and HDL cholesterol). Hormonal profile evaluation was performed by chemiluminescence immunoassay (CLIA) [Growth Hormone (GH), Insuline-like Growth Factor 1 (IGF-1), Thyroid Stimulating Hormone (TSH), Follicle- Stimulating Hormone (FSH), Luteinising Hormone (LH), Adrenocorticotropic Hormone (ACTH), cortisol, prolactin].

### 2.3. Assessment of Chronotype 

At the study entry, subject Morningness-Eveningness in both patients and controls was measured with the Horne–Ostberg Morningness-Eveningness Questionnaire (MEQ) [30]. The MEQ included 19 questions about preferred sleep time and daily performance. The scores ranged from 16 to 86. Individuals, based on their scores, were categorised as being a morning (59–86), neither or intermediate (42–58), or evening (16–41) chronotype. 

### 2.4. Sample Size Justification and Power

Considering 2 independent study groups (patients with CP vs. control group), the sample size was calculated using Sample Size Calculator Clinical Calc (https://clincalc.com/stats/samplesize.aspx, accessed on 24 September 2021), as previously reported in other studies [31,32]. In detail, the sample size was calculated by the differences of mean ± standard deviation (SD) of the chronotype score in patients with CP and control group (47.8 ± 12.6 vs. 57.6 ± 9.5 score, respectively). Considering a type I (alpha) error of 0.10 and a type II (beta) of 0.10 and a power size of 95%, the number of cases required in patients with CP and control group were 28 for each group.

### 2.5. Statistical Analysis 

The data distribution was determined by a Kolmogorov–Smirnov test. Data not normally distributed were normalised by logarithm. Results were described as mean ± standard deviation (SD) or percentage/number. Fisher’s exact text was used to determine associations between gender, BMI and chronotype categories. Analysis of variance (ANOVA) test followed by the Bonferroni post hoc test was used to analyse the differences among chronotypes between-group. A partial correlation was performed to control the effect of sex as a confounding factor on the chronotype score. Considering the whole population of the study (patients and controls), in the logistic analysis, a proportional odds ratio (OR) model, *p*-value, 95% interval confidence (IC) and R^2^, was performed to assess the association between chronotype score and presence/absence of craniopharyngioma. 

*p* values < 0.05 were considered statistically significant. Data were analysed using the SPSS Software (PASW Version 21.0, SPSS Inc., Chicago, IL, USA) and MedCalc^®^ package (Version 12.3.0 1993–2012 MedCalc Software bvba-MedCalc Software, Mariakerke, Belgium).

## 3. Results

### 3.1. Demographic and Anthropometric Parameters

The clinical characteristics of patients and controls are shown in Table 2. Patients and controls were not significantly different for gender, age and BMI. In particular, obesity was present in all the enrolled subjects, but there were no differences in the distribution of grade I or II obesity between patients and controls (*p* = 0.560). On the contrary, WC was significant higher in patients than in controls (*p* = 0.01).

### 3.2. Metabolic Parameters and Blood Pressure

The metabolic parameters of patients and controls are shown in Table 2. Plasma glucose, total and LDL cholesterol, triglycerides, SBP and DBP were significantly higher in the patient group than in the controls (plasma glucose: *p* = 0.015; total cholesterol: *p* = 0.006; LDL cholesterol: *p* = 0.007; triglycerides: *p* = 0.003; SBP: *p* = 0.006; DBP: *p* = 0.021, while HDL cholesterol was significantly lower in the patients than in controls (*p* = 0.023). 

### 3.3. Chronotype Categories

**Figure 1:**
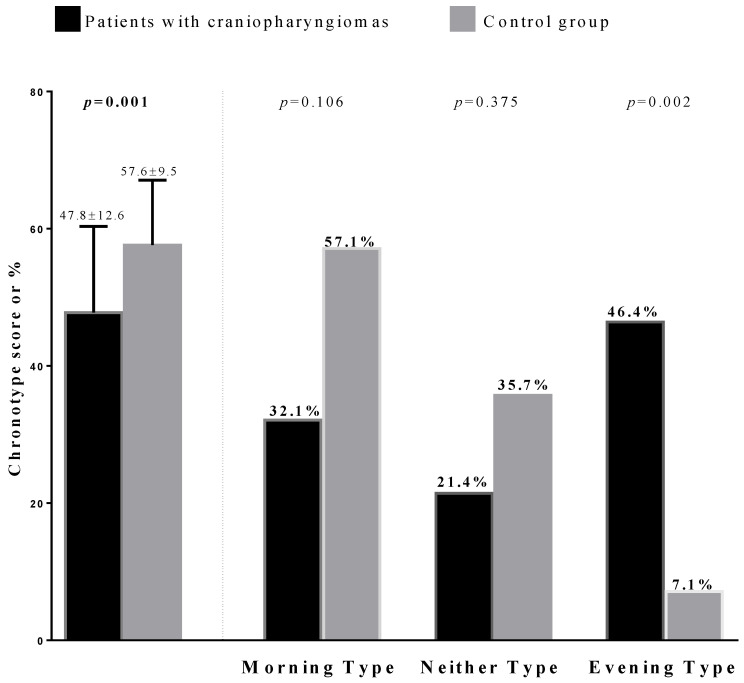
Prevalence in chronotype categories in patients and controls. Data are reported as Mean ± SD and percentage; *p* values < 0.05 were considered statistically significant.

In the patient group, 9 patients (32.1%) exhibited the morning chronotype, 6 (21.4%) the intermediate chronotype and 13 (46.4.%) the evening chronotype (Figure 1, Table 3). No significant differences were found in gender and age among the chronotype categories.

Patients with the evening chronotype had higher blood pressure values and worse metabolic parameters than those with the morning chronotype except for total and HDL cholesterol and triglycerides (Table 3). In the control group, 16 (57.1%) subjects exhibited the morning chronotype, 10 (35.7%) the intermediate chronotype and only 2 (7.1%) the evening chronotype (Figure 1, Table 4). The prevalence of intermediate and evening chronotypes was higher in females than males (*p* = 0.037; Table 4), while males had a higher prevalence of morning chronotype. No significant difference was found in age, blood pressure, LDL and HDL cholesterol among chronotype categories (Table 4). However, subjects with evening chronotypes had higher BMI and WC and exhibited worse metabolic parameters than those with the morning chronotype, as shown in Table 4.

### 3.4. Correlation Studies

Correlation analysis adjusted for sex was performed to assess the association of the chronotype score with age, anthropometric and metabolic parameters in both patients and controls. 

In patients, the chronotype score was inversely correlated to BMI (r = −0.836, *p* = 0.000), WC (r = −0.676, *p* = 0.000), SBP (r = −0.490, *p* = 0.010), DBP (r = −0.384, *p* = 0.042), plasma glucose (r = −0.502, *p* = 0.008), total cholesterol (r = −0.378, *p* = 0.05), LDL cholesterol (r = −0.432, *p* = 0.024), triglycerides (r = −0.398, *p* = 0.040) and positively correlated with HDL cholesterol (r = 0.551, *p* = 0.003) (Table 5). No correlation was found between age and chronotype (r = 0.023, *p* = 0.909). 

In controls, the chronotype score was inversely correlated to BMI (r = −0.654 *p* = 0.000), WC (r = −0.563, *p* = 0.002), plasma glucose (r = −0.745, *p* = 0.000), total cholesterol (r = −0.551, *p* = 0.003), LDL cholesterol (r = −0.501, *p* = 0.008) (Table 6). No correlation was found among chronotype and age, blood pressure, triglycerides, HDL cholesterol.

Considering the whole population of the study (patients and controls), a logistic regression analysis was performed to assess the association between chronotype score and presence/absence of craniopharyngioma. In this model, the chronotype score was significantly associated with the presence of craniopharyngioma. (OR  =  0.93, *p*  =  0.004, 95% CI 0.88–0.98, R^2^  =  0.16).

## 4. Discussion

Based on our present knowledge, this is the first study performed to evaluate the association among CP, chronotypes and metabolic risk profile. In particular, our results showed that, compared to age and BMI-matched controls, patients with CP had a higher prevalence of the evening chronotype, which was associated with a greater impairment of their metabolic profile. 

Obesity is one of the main concerns of patients with CP. Together with hypopituitarism, obesity and the correlated metabolic alterations are common findings in these patients, worsen their quality of life and lead to a higher risk of morbidity and mortality [3,33,34,35]. Thus, CP patients require an appropriate long-term follow-up not only to rule out recurrences and to ensure a proper endocrine replacement, but also to achieve the control of metabolic factors influencing their cardiovascular risk [16,36].

In the pathogenesis of obesity in the CP patients, the tumour infiltration and/or the surgical treatment of the hypothalamic areas projecting their hunger- and satiety-related signals or controlling the sleep-wake regulatory system [37] and the circadian rhythm synchronisation, are considered key factors [8,12,38]. Unfortunately, the hypothalamic obesity is mainly resistant to conventional weight-reducing therapies and this in part due to its association to multiple neuroendocrine deficits, which require adequate hormone substitution. In our study, however, all patients were replaced with hormonal therapies and showed an appropriate hormonal assessment, leading us to hypothesise that they were receiving appropriate replacement treatments. 

Lifestyle modification, which involves altering and maintaining long-term habits that include being physically active and healthy eating, remains the treatment of choice [39], although with poor and short-term results in any case [40]. This is especially true in patients with CP and other hypothalamic obesity syndromes, as they are poor responders to dietary and lifestyle modifications [8,41,42,43] and often show marked weight gain even after forced caloric restriction, suggesting that hyperphagia is not the central problem. Chronotype and circadian rhythms are emerging risk factors of obesity and cardiometabolic diseases [21,22]. Several genes, commonly termed clock genes, represent the molecular mechanism underlying the circadian functions [44]. The biological rhythms in humans present large interindividual differences that determine their chronotype or circadian typology, depending on different factors, such as latitude, work schedule and social engagement [45]. In addition, a clear gender difference has been reported in chronotype, as women, especially young ones, have on average more morningness typology than males [46], although this difference diminishes with age [47].

Belonging to a chronotype rather than another seems to affect the health profile. In fact, growing evidence suggests that the evening chronotype tends to eat fewer, larger and unwholesome meals [21,48], probably also linked to poor sleep quality [49] or lower self-control levels [21,50]. In particular, there is consolidated evidence linking sleep deprivation, eating times and obesity [51]. Similarly, sleep disturbances and daytime sleepiness are commonly found in patients with CP [37]. Nevertheless, no studies have been focused on the association among CP, chronotypes and altered metabolic profile. In our observational case-control, patients with CP were at more risk of having higher chronotype score as compared with controls. In particular, patients with CP showed a higher prevalence of the evening chronotype (46.4%), while among the controls the morning chronotype was most prevalent (57.1%). No significant difference was found in gender among chronotype in patients with CP. Although with some differences, both patients and controls with the evening chronotype exhibited a greater impairment of their metabolic profile than those with the morning chronotype. In this context, despite the absence of significant differences in BMI, patients with CP presented a worse metabolic profile and higher blood pressure compared to controls. This is in line with the observations of the study by Ferraù et al. [52] in which patients with CP, both adults and children, showed increased values of the Visceral Adiposity Index, a surrogate marker of cardiovascular risk [53]. Data on the influence of the chronotype on the metabolic profile in the CP setting are lacking, but our results seem to be in line with the available literature on obese people without CP, which indicates that evening or late chronotype was associated with a greater degree of misalignment between internal timing and external conditions [5,54,55], potentially leading towards disruptions in central and peripheral rhythms [56]. Of interest, the higher prevalence of the evening chronotype among patients with CP as compared with BMI-matched controls, associated with their worse metabolic profile, let us to speculate that the injury of the hypothalamic area controlling not only the feeding behaviour, but also the sleep-wake regulatory system and in circadian rhythm synchronisation, might represent an adjunctive risk for CP patients and a possible target for their integrate management. This hypothesis is in line with the possible bidirectional relationship between circadian rhythms and diseases [57]. To the best of our knowledge, this is the first study performed on adult patients with CP that reported the significant association of the evening chronotype with CP and the metabolic profile in this setting. Considering the well-established crucial role of specific dietary behaviour to develop personalised chrono-nutrition interventions [58], our study supports the relevance of the evaluation of the chronotype for both weight and metabolic management in patients with CP to better facilitate adherence to personalised nutrition interventions and adds a contribution to the growing literature on the interaction between chronotypes and metabolic health. This could be of particular relevance given the scant results of the available therapeutical options in patients with CP. However, there are some limitations to this study that need to be addressed as well. Firstly, the observational case-control design does not allow to draw conclusions on causal or temporality inferences of the association between the chronotype and the metabolic profile of patients with CP. Secondly, the sample size is relatively small, due to the rarity of the disease, and the recruitment was conducted in single centre, as well as inclusion/exclusion criteria in our study were stringent. This limitation, however, contributed to make a well-characterised population and the enrolment in a single centre ensured homogeneity in patients’ assessment, treatment and follow-up modalities. In addition, it is important to highlight that many factors that influence the chronotype, such as nutrition, latitude, work schedule, sleep habits and social engagement, were not included in this study. In particular, recovery of sleep dysfunction could help to maximise adherence to life-style interventions. However, there has been no screening or guidelines for the treatment of sleep disturbances in these patients [37]. Lastly, in this study abdominal adiposity, which is considered the major contributor to cardiovascular risk and metabolic syndrome [59], was measured clinically by WC and not by gold standard instrumental techniques, such as computed tomography scans or magnetic resonance imaging. Similarly, the study lacks the assessment of target organ damage to predict the cardiovascular risk. However, this is the first study that suggests to evaluating the chronotype in the management of obesity in patients with CP, which implied potential applications on the therapeutic approach and the cardiometabolic health of these patients. All these aspects deserve further investigation on a larger scale of patients to understand the relationship between circadian typology, obesity and metabolic alterations in patients with CP. 

## 5. Conclusions

In conclusion, for the first time thus far our study puts the light on the association of the CP with chronotypes and metabolic alterations in this disease, which are the main determinants of the reduced quality of life, higher morbidity and mortality in this setting of patients. This novel finding suggests that alterations of chronotype might represent an adjunctive risk for CP patients and a possible target for their integrate management, which is still lacking satisfactory treatments. Our findings may provide a focus for future clinical care and research. 

## Figures and Tables

**Table 1:** Craniopharyngioma patients’ characteristics at study entry.

Parameters	Craniopharyngioma Patients *n* = 28
**Craniopharyngioma treatment**	
Neurosurgery only	16
Neurosurgery + Radiotherapy	12
**Pituitary hormone deficiency** At least one deficit	
GH	20
Gonadotropins	18
TSH	20
ACTH	25
Central diabetes insipidus	23
Panhypopituitarism	18
rGH replacement therapy	16

**Abbreviations: GH**, Growth Hormone; **TSH**, Thyroid Stimulating Hormone; **ACTH**, Adrenocorticotropic Hormone; **rGH**, recombinant Growth Hormone. Data are reported as the number of patients with specific pituitary hormone deficiency.

**Table 2:** Differences in study parameters between patients with craniopharyngioma and control group.

Parameters	Craniopharyngioma Patients *n* = 28	Control Group *n* = 28	*p*-Value
**Demographic characteristics**			
Gender (Males)	13 (46.4%)	13 (46.4%)	0.205
Age (Years)	42.6 ± 15.8	46.5 ± 12.9	0.355
**Anthropometric measurements**			
Weight (kg)	95.4 ± 15.5	94.7 ± 17.8	0.859
Height (m)	1.7 ± 0.08	1.6 ± 0.11	0.568
BMI (kg/m^2^)	34.3 ± 3.5	34.7 ± 2.6	0.574
Grade I obesity (*n*, %)	15 (53.6%)	15 (53.6%)	0.560
Grade II obesity (*n*, %)	13 (46.4%)	13 (46.4%)	
WC (cm)	106.1 ± 10.1	99.2 ± 10.6	**0.011**
**Blood pressure**			
SBP (mmHg)	121.9 ± 12.8	114.8 ± 11.2	**0.022**
DBP (mmHg)	77.8 ± 9.0	73.5 ± 10.5	**0.003**
**Metabolic profile**			
Plasma glucose (mg/dL)	101.8 ± 13.1	95.2 ± 7.5	**0.015**
Total cholesterol (mg/dL)	207.3 ± 47.0	182.9 ± 33.7	**0.006**
LDL cholesterol (mg/dL)	135.7 ± 44.6	111.7 ± 38.4	**0.007**
HDL cholesterol (mg/dL)	40.7 ± 9.4	46.2 ± 9.6	**0.023**
Triglycerides (mg/dL)	154.3 ± 48.9	124.9 ± 35.1	**0.003**

Data are reported as Mean ± SD and number/percentage; *p* values < 0.05 were considered statistically significant. **Abbreviations: BMI**, body mass index; **WC**, waist circumference; **SBP**, systolic blood pressure; **DBP**, diastolic blood pressure; **HDL**, high-density lipoprotein; **LDL**, low-density lipoprotein.

**Table 3:** Differences in study parameters of patient with craniopharyngioma according to chronotype categories.

Parameters	Morning Type *n* = 9, 32.1%	Neither Type *n* = 6, 21.4%	Evening Type *n* = 13, 46.4%	F-Value	*p*-Value
**Gender**					
Males (*n*, %)	5 (55.6%)	3 (50.0%)	5 (38.5%)		0.885
Females (*n*, %)	4 (44.4%)	3 (50.0%)	8 (61.5%)		
**Age (years)**	41.7 ± 12.3	55.3 ± 15.8	37.5 ± 15.7	3.06	0.063
**Anthropometric measurement**					
BMI (kg/m^2^)	31.2 ± 1.2	32.0 ± 1.7	37.5 ± 1.9 ^a,b^	42.75	**0.000**
Grade I obesity (*n*, %)	9 (100.0%)	5 (83.3%)	1 (7.7%)		0.000
Grade II obesity (*n*, %)	0 (0.0%)	1 (16.7%)	12 (92.3%)		
WC (cm)	100.9 ± 8.4	102.3 ± 6.5	111.5 ± 10.3 ^a^	4.29	**0.037**
**Blood pressure**					
SBP (mmHg)	114.4 ± 8.8	116.2 ± 15.2	129.6 ± 9.9 ^a,b^	6.22	**<0.05**
DBP (mmHg)	73.9 ± 5.5	73.0 ± 8.4	82.7 ± 9.2 ^a,b^	5.43	**<0.05**
**Metabolic profile**					
Glycemia levels (mg/dL)	95.2 ± 8.2	95.8 ± 12.3	109.2 ± 12.9 ^a,b^	4.94	**<0.05**
Total cholesterol (mg/dL)	193.3 ± 24.0	174.3 ± 47.5	232.1 ± 47.8 ^b^	4.68	**0.029**
Triglycerides (mg/dL)	142.3 ± 35.9	117.2 ± 52.4	179.6 ± 43.4 ^b^	4.80	**0.021**
LDL cholesterol (mg/dL)	117.4 ± 18.5	108.9 ± 41.6	160.8 ± 47.3 ^a,b^	5.06	**<0.05**
HDL cholesterol (mg/dL)	47.4 ± 9.1	42.0 ± 10.7	35.4 ± 5.4 ^b^	6.23	**0.005**

Data are reported as Mean ± SD and number/percentage; frequencies were analysed by Fisher’s exact text. Differences in the three groups were analysed by one way ANOVA test with the Bonferroni test as post-hoc test. ^a^ *p* values vs. morning type, ^b^ *p* values vs. neither type. *p* value < 0.05 were considered statistically significant. **Abbreviations: BMI**, Body Mass Index; **WC**, waist circumference; **SBP**, systolic blood pressure; **DBP**, diastolic blood pressure; **HDL**, high-density lipoprotein; **LDL**, low-density lipoprotein.

**Table 4:** Differences in study parameters of control group according to chronotype categories.

Parameters	Morning Type *n* = 9, 32.1%	Neither Type *n* = 6, 21.4%	Evening Type *n* = 13, 46.4%	F-Value	*p*-Value
**Gender**					
Males (*n*, %)	11, 68.8%	2, 20.0%	0, 0%		0.885
Females (*n*, %)	5, 31.3%	8, 80.0%	2, 100%		
**Age (years)**	47.31 ± 16.44	44.40 ± 17.01	50.50 ± 6.36	0.16	1.000
**Anthropometric measurement**					
BMI (kg/m^2^)	33.42 ± 1.95	35.93 ± 2.69	38.70 ± 0.72 ^a^	7.46	**0.012**
Grade I obesity (*n*, %)	12, 75.0%	3, 30.0%	0, 0%		0.000
Grade II obesity (*n*, %)	4, 25.0%	7, 70.0%	2, 100%		
WC (cm)	96.38 ± 9.82	99.60 ± 7.94	120.00 ± 0.00 ^a,b^	6.17	**<0.05**
**Blood pressure**					
SBP (mmHg)	115.31 ± 11.61	114.70 ± 12.23	112.50 ± 3.53	0.05	1.000
DBP (mmHg)	72.19 ± 10.33	73.80 ± 11.54	82.50 ± 3.53	0.08	0.620
**Metabolic profile**					
Glycemia levels (mg/dL)	92.38 ± 7.11	97.10 ± 4.41	108.50 ± 7.77 ^a^	6.51	**0.007**
Total cholesterol (mg/dL)	169.00 ± 22.29	194.70 ± 38.34	234.50 ± 14.84 ^a^	5.86	**0.017**
Triglycerides (mg/dL)	120.06 ± 27.19	119.80 ± 38.31	189.00 ± 12.72 ^a,b^	4.52	**<0.05**
LDL cholesterol (mg/dL)	96.55 ± 32.16	127.34 ± 40.13	154.20 ± 13.01	4.03	0.109
HDL cholesterol (mg/dL)	48.44 ± 9.70	43.40 ± 9.81	42.50 ± 0.70	1.02	1.000

Data are reported as Mean ± SD and number/percentage; frequencies were analysed by Fisher’s exact text. Differences in the three groups were analysed by one way ANOVA test with the Bonferroni test as post-hoc test. ^a^ *p* values vs. morning type, ^b^ *p* values vs. neither type. *p* value < 0.05 were considered statistically significant. **Abbreviations: BMI**, body mass index; **WC**, waist circumference; **SBP**, systolic blood pressure; **DBP**, diastolic blood pressure; **HDL**, high-density lipoprotein; **LDL**, low-density lipoprotein.

**Table 5:** Correlations of chronotype score with study parameters adjusted for sex in patients with craniopharyngioma.

	Simple Correlations	
Parameters	r	*p*-Value
**Age (years)**	0.023	0.909
**Anthropometric measurement**		
BMI (kg/m^2^)	−0.836	**0.000**
WC (cm)	−0.676	**0.000**
**Blood pressure**		
SBP (mmHg)	−0.490	**0.010**
DBP (mmHg)	−0.394	**0.042**
**Metabolic profile**		
Glycemia levels (mg/dL)	−0.502	**0.008**
Total cholesterol (mg/dL)	−0.378	**0.050**
LDL cholesterol (mg/dL)	−0.432	**0.024**
HDL cholesterol (mg/dL)	0.551	**0.003**
Triglycerides (mg/dL)	−0.398	**0.040**

*p* values < 0.05 were considered statistically significant. **Abbreviations: BMI**, body mass index; **WC**, waist circumference; **SBP**, systolic blood pressure; **DBP**, diastolic blood pressure; **HDL**, high-density lipoprotein; **LDL**, low-density lipoprotein.

**Table 6:** Correlations of chronotype score with study parameters adjusted for sex in the control group.

	Simple Correlations	
Parameters	r	*p*-Value
**Age (years)**	0.075	0.710
**Anthropometric measurement**		
BMI (kg/m^2^)	−0.654	**0.000**
WC (cm)	−0.563	**0.002**
**Blood pressure**		
SBP (mmHg)	0.039	0.846
DBP (mmHg)	−0.108	0.592
**Metabolic profile**		
Glycemia levels (mg/dL)	−0.745	**0.000**
Total cholesterol (mg/dL)	−0.551	**0.003**
LDL cholesterol (mg/dL)	−0.501	**0.008**
HDL cholesterol (mg/dL)	0.291	0.141
Triglycerides (mg/dL)	−0.302	0.125

*p* values < 0.05 were considered statistically significant. **Abbreviations: BMI**, body mass index; **WC**, waist circumference; **SBP**, systolic blood pressure; **DBP**, diastolic blood pressure; **HDL**, high-density lipoprotein; **LDL**, low-density lipoprotein.

## Data Availability

Not applicable.

## Abbreviations

CP	craniopharyngioma
BMI	body mass index
WC	waist circumference
